# Hawes' Tongue and Duct Compressor

**Published:** 1867-07

**Authors:** 


*Hawes' Tongue and Duct Compressor.*.—Since the attention of the profession was first directed to the adhesive property of gold, and the manner of using it in this form, a number of appliances have been invented to overcome the trouble arising from the flow of saliva and the movements of the tongue. None of them, however, appear to answer the purpose so well as the simple appliance of Dr. G. W. Hawes, so modified by Dr. W. N. Morrison as to render it an indispensible instrument in filling the inferior teeth.

By the use of this instrument the movements of the tongue are arrested, the flow of saliva checked, and the operations upon the inferior molars and bicuspids readily performed, without the annoyance to the patient which results from filling the mouth with napkins. "With this instrument the tongue may be clamped down in place and kept in position as long as desired." "The sublingual and submaxillary ducts may be very effectually closed by placing upon them rolls or pads of bibulous paper before applying the compress; a pad of paper or cloth should also be placed on the tongue before applying the instrument." It is so effectual in accomplishing the purpose for which it is designed, that we feel no hesitation in recommending it to the profession as the most useful instrument of the kind which has ever been invented.

